# The Neurobiological Basis of the Conundrum of Self-continuity: A Hypothesis

**DOI:** 10.3389/fpsyg.2022.740542

**Published:** 2022-05-18

**Authors:** Morteza Izadifar

**Affiliations:** Institute of Medical Psychology and Human Science Center, Ludwig-Maximilian University Munich, Munich, Germany

**Keywords:** self-continuity, identity, time, the principle of reafference, the corollary discharge principle, discrete conscious perception

## Abstract

Life, whatsoever it is, is a temporal flux. Everything is doomed to change often apparently beyond our awareness. My body appears totally different now, so does my mind. I have gained new attitudes and new ambitions, and a substantial number of old ones have been discarded. But, I am still the same person in an ongoing manner. Besides, recent neuroscientific and psychological evidence has shown that our conscious perception happens as a series of discrete or bounded instants—it emerges in temporally scattered, gappy, and discrete forms. But, if it is so, how does the brain persevere our self-continuity (or continuity of identity) in this gappy setting? How is it possible that despite moment-to-moment changes in my appearance and mind, I am still feeling that I am that person? How can we tackle with this second by second gap and resurrection in our existence which leads to a foundation of wholeness and continuity of our *self*? How is continuity of self (collective set of our connected experiences in the vessel of time) that results in a feeling that one’s life has purpose and meaning preserved? To answer these questions, the problem has been comprehended from a philosophical, psychological, and neuroscientific perspective. I realize that first and foremost fact lies in the temporal nature of identity. Having equipped with these thoughts, in this article, it is hypothesized that according to two principles (the principle of reafference or corollary discharge and the principle of a time theory) self-continuity is maintained. It is supposed that there should be a precise temporal integration mechanism in the CNS with the outside world that provides us this smooth, ungappy flow of the *Self*. However, we are often taken for granted the importance of self-continuity, but it can be challenged by life transitions such as entering adulthood, retirement, senility, emigration, and societal changes such as immigration, globalization, and in much unfortunate and extreme cases of mental illnesses such as schizophrenia.

## The Enigma: The Paradox of Personal Persistence and Its Disrupted Nature

Life, whatsoever it is, is a temporal flux. Everything is doomed to change often apparently beyond our awareness. My body appears totally different now, so does my mind. I have gained new attitudes and new ambitions, and a substantial number of old ones have been discarded. But, I am still the same person in an ongoing manner. How is it possible that despite moment-to-moment changes in my appearance and mind, I am still feeling that I am that person? How can we tackle with this second by second gap and resurrection in our existence which leads to a foundation of wholeness and continuity of our *self*? How is continuity of self (collective set of our connected experiences in the vessel of time) that results in a feeling that one’s life has purpose and meaning preserved? How can we explain the paradox of continuous change and permanence, while having the perception of being as we are, and yet changed?

**Figure 1:**
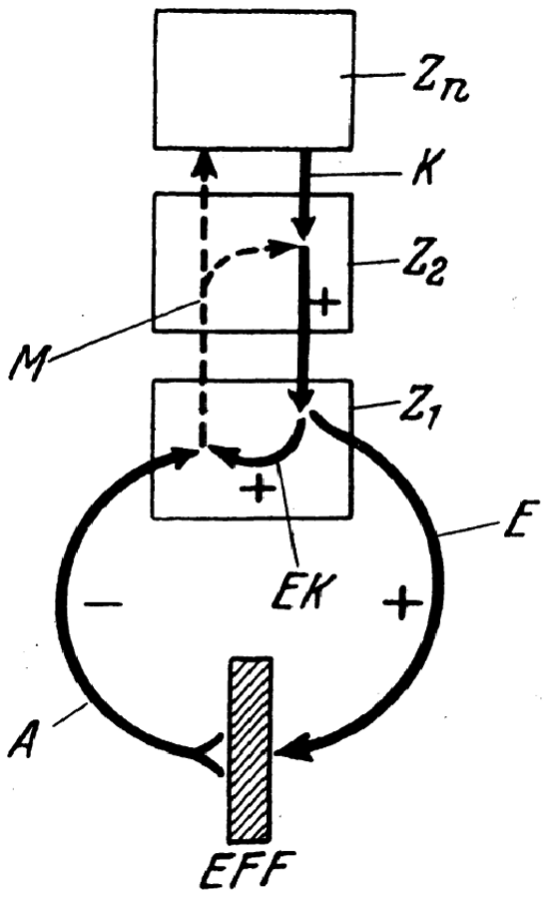
General schema for the principle of reafference. From a higher center Zn a command K is sent to a lower center (Z2); from there a neuronal command is given to an executive center (Z1), which in turn innervates (by an efference E) an effector (EFF). Simultaneously, a copy of the efferent command E is stored [efference copy (EK)]. After the movement, an afferent signal A (or the “reafference”) is sent to Z1, where this reafferent signal is compared with the EK. If A (with a minus sign) resembles to EK (with a plus sign), both signals cancel each other, and the sensorimotor cycle is completed. If EK and A do not match, a message (M) is sent to Z2 and Zn to correct or to re-initiate the sensorimotor cycle until it ultimately comes to an end. Reproduced from von Holst and Mittelstaedt (1950/1971), with permission from Elsevier.

At first, the concept of self and continuity are clarified and then self-continuity is explained in a fine-grained analysis.

## Self and Continuity

What is self? The self is the main constituent of consciousness. It can be designated as “backbone of our existence” (Sadeh and Karniol, 2012). From some thinkers’ view, the self (James, 1890/1998; Mead, 1934), or psychosocial identity (Erikson, 1968) are comprised of two types. It can be reflectively established as having certain characteristics (James’ Me or self-as-object), and it can be established in a pre-reflective sense of familiarity with one’s body, opinions, and actions or the self as observer (James’ I or self-as-subject), characterized as subjective sense of identity by Erikson (1968). For a consistent sense of self (James) or psychological identity (Erikson), both a synchronic integration of present features across different circumstances and a diachronic integration over time—self-continuity—are fundamental. In its diachronic phase, the self is the same over time, and it is felt as continuous.

Concept of *self* has been also proposed by neuroscientists in the modern era. Most of studies have shown that the brain activation in the medial regions of cortex during self-related stimuli. The activation in these so-called cortical midline structures (CMS) has occurred across all functional realms such as verbal, spatial, emotional, and facial (Kelly et al., 2004; Mitchell et al., 2008; Zhu et al., 2012). Although plentiful studies have confirmed the role of (CMS) in creating the self, however, neural basis of the self is still obscure. Likewise, much more obscure is the brain (neuronal) mechanism in persisting the continuity of self.

Having said about the concept of self, what is continuity? In this article, what is meant about continuity is straightforward. In a very common and broad sense, the word “continuity” has been used. It can be described as the absence of gaps which is said to be continuous. In other words, it is composed of a very smooth, sleek, and uninterrupted sense.

Then, what is self-continuity? Why is it vital for our existence? I suppose that *self-continuity*, or *diachronic self*, or *continuity of identity, or phenomenal continuity, or psychological continuity* (the terms that explain this concept are diverse and the literature is extremely confusing) is the combination of the many life threads or life stories which are bound together in the tube of time. To put it in a different form, the sense of self-continuity refers to unique and persisting mechanism that outline who we are and condenses an individual’s past and present self, and connects all to a self who he or she will become. As if there is a backbone of the self that connects the individual in the past, present, and future projects together into an ongoing sense of personal identity.

Furthermore, these stories or threads are predisposed by cultural values or norms, and thus “sense of self” is recreated between people in terms of one’s connection with others which contribute to a sense of coherence, stability, and continuity of self—the persistence of threads of meaning in life. However, serious illnesses such as schizophrenia disrupts these biographical threads of meaning and leads to the sense of identity problem in which people also claim that their life in the world has changed drastically.

Why is self-continuity important for our existence? What are its advantages? To answer these questions, we should notice to the fact that we are inclined to persist until we cease—our most basic selfish desires are perseverance. As we say, we want to live, to have a determination to move on. The sense of self-continuity is central in construing and cognitively forming our social world, controlling our emotional responses to that world, and instructing our behavior in light of both—our survival. Besides, for each of us, conscious of who we are, where we have come from, and sensation of an inner self-continuity across time is fundamental to an individual wellbeing. We are often taken for granted the importance of self-continuity, but it can be challenged by life transitions such as entering adulthood, retirement, senility, emigration, and societal changes such as immigration, globalization, and in much unfortunate and extreme cases of mental illnesses such as schizophrenia.

Persistence of identity across time plays a leading role in survival and supports the fact that personal identity is deeply important. Research implies that the faculty to recognize the self is an evolutionary adaptation that is effortlessly persistent through automatic systems (Parfit, 1971; Hirsch, 1976; Wiggins, 1980/2001; Damasio, 2011). In early childhood, individuals first understand “I am” and then understand “I am and I was (about age five).” Their past self and present selves become connected as they first begin to develop a personal timeline (Fivush, 2011). In other words, I assume that any organism needs a continuous, chronological awareness of self simply to consolidate incoming information and execute daily behaviors. For example, one experiences a pleasure or pain, or intends an action, only if that pleasure or pain experienced and processed by the brain which belongs to that *self*, or that intention shaped, by one of the interconnected moments of consciousness that construct that person. Preserving a sense of self-continuity is initial to human activities. Yet, how do we do it?

There are wealthy of studies that indicate self-continuity has numerous positive consequences for psychological functioning and wellbeing (Bluck and Alea, 2008; Sani et al., 2008). Perceiving a sense of self-continuity over time and across different situations can help individuals feel grounded in a social and cultural context, and accordingly manage their existential fears (Landau et al., 2008). However, sometimes the procedure of self-continuity does not function as it is expected. For example, in the case of identity crisis, immigrants who suffer from a disruption in identity continuity may experience the remoteness between them and their source culture. Thus, this disruption of self brings lots of psychological problems for them.

Furthermore, what distresses people in thinking that their personalities not change too much or too rapidly. Persons identify themselves with their beliefs, objectives, and character traits and feel that the steadiness of these is somehow vital to remaining the same person. Moreover, that psychological persistence is required to be caused by the constant existence of the same performing brain. Besides, the feeling of an individual as a continuing subject makes possible other psychological activities such as self-regarding emotions and self-interpretation.

To reassure ourselves of the value of preserving self-continuity, a simple *Gedankenexperiment* suffices. What if I woke up not knowing that I was me? Imagine that it would totally disrupt everything from daily activities and personal relationships, to future plans and goals. Or, for seconds look at the wall, then again change your look at somewhere else. The image changed but the content of conscious state not. The content is still steady, the same, and continuous. You are still the person who looks. Changing perspective from the wall to somewhere else did not make you another person.

Having attended to the importance of self-continuity, you may wonder whether it is possible that this thread of identity is torn. Here are real examples of the fragmented, unprocessed moments of experience that exist when self-continuity no longer functions: schizophrenics are not entirely self-conscious, nor are they proficient of many of the sorts of experiences which “normal” persons have (Freud, 1939). They do not yet have the cognitive ability to link different moments and features of experience into more multifaceted wholes. Or, consider the situation of a senile person. A senile person is incompetent to remember who and what he is, what he has made through his past life, what roles he has performed in the lives of others. He cannot get the story straight on who she is, and so lives in bewilderment about himself. These deficits make it difficult to form a coherent self-conception and self-continuity. Furthermore, people who are in persistent vegetative circumstances or with advanced dementia, certainly are not the same people they once were. Their family may well say of them “She is no longer there.”

Such accounts echo a painful understanding that the persons’ self-continuity (or identity) has been altered so severely as to make them almost strange as the persons they have known. By considering these cases we realize that self-continuity is necessary for maintaining personal identity because personal identity and self-continuity cannot fall apart: because all the experiences in a single stream of consciousness are simultaneous. However, it is worth noting that sometimes people can suffer from amnesia, character change, and illusory memories while maintaining their identity. In cases where a person suffers a loss of identity (maybe due to a loss of all previous memories), while still enjoying an uninterrupted, smooth stream of consciousness. However, there is a puzzle with this kind of self-continuity which has been ignored.

It should be added that continuity of self is a dynamic and evolutionary concept. Like continuity in a stage play, continuity is also evolving over a lifetime of action and learning and battle and joyfulness and heartbreak. All of our experiences weave into the network of one’s conception of oneself over time. Furthermore, this necessitates that the effects of past experiences and the anticipation of future ones mingle with one’s present experience. It seems that according to this view each time a person experiences something, she consciously deliberates the past and future and decides how to comprehend the present on that basis.

Let me turn back to that old and good question, to that paradoxical state of our existence: How does our inherent fragmentary, patchy consciousness like pearls of necklace links together and we recognize ourselves every morning in the mirror? Many of great thinkers have tried to answer this question. However, their answers seem to me not convincing. I presume that the puzzle is far from our intellectual speculations. Before introducing my hypothesis, let me present a brief historical review and also new findings about this enigmatic question of our life.

## The Same and the Same Enigma: Different Readings of How Self-Continuity Is Preserved

All beings must be continuous, although its nature is discrete. Consciousness is not an exception in this realm, so is our identity. Our experiences originate in a perceptual and mental flow, although it is rather as a series of discrete or bounded instants. The brain is highly plastic rather than rigid, and even our own brain continually alters its cells and activity configurations. Such a continuous view of identity is very unlikely due to the continuous change of both psychological and physiological quality. Thus, if consciousness and also our body structure emerges in temporally scattered, gappy and discrete forms, how does the brain persevere our self-continuity (or continuity of identity)? When I wake up, each morning, how do I know that I am “me”? Over days, weeks, and also a lifetime, how can people develop a sense of self-continuity? How is it possible that, from the beginning of childhood, we accumulate the events of our lives and then incorporate a lifetime of experiences into a biographical identity or life story? How could it be that individuals are able to consciously work to create, remodel, and rebuild a life story over time from the recalled acts of their lives? Taken in a philosophical sense, how is it possible that, while both our cognitive and physiological qualities change over time, we are one and the same person over time? How is our “self” enduring over time?

To answer these questions, if one sees the problem from a psychological and neuroscientific perspective, one realizes that first and foremost fact lies in the temporal nature of identity. Furthermore, the identity is context-independent, from the temporary changes in our psychological contents as they are related to changes in context. For instance, the identity which I preserve with my mother is completely different from the identity which I have with my girlfriend. I believe, however, that over time, I have a consistent mental picture of myself, that is, in a diachronic sense that amounts to “self-continuity,” although it seems that identity remains an abstract term albeit to some extent.

The conundrum of self-continuity is a very old concept which has been deliberated in a broad spectrum of thinkers dating back to the comments by Aristotle that “the living thing has a shift concept and remains unchanged because certain temporally extended things are divisible” (cited in Wiggins, 1980/2001). Afterward, lots of scholars endeavored to untangle the complexities of this phenomenon. There exists a wealthy number of philosophers, psychologists, and neuroscientists who are on the top of the list. A very long list of philosophers in 20^th^ century (Cassirer, 1923; von Bertalanffy, 1968; Parfit, 1971; Perry, 1976; Rorty, 1976; von Hirsch, 1976; MacIntyre, 1977), and touchstone psychological theorists (James, 1902; Erikson, 1968; Piaget, 1968), and contemporary (Chandler et al., 2003; Pöppel, 2010; Vignoles, 2018) have all tried to come to grasp the question of self-persistence in time as a significant feature of personal identity and also as a “universal in the human experience”(Levine and Thompson, 2004).

Let us begin with philosophers’ standpoint about the question of self-continuity. Philosophers’ arguments on the conundrum of self-continuity can be summarized into two main groups of answer: those that can be categorized as giving *physical criteria* and those as giving *psychological criteria*.

Among some philosophers who are in the camp of *physical criteria*, it is argued that generally self-continuity is tied to spatiotemporal bodily continuity and it is a necessary condition for personal identity (Shoemaker and Swinburne, 1984). Its basic form is as the following: person A in *t*, is the same with person B in t, if and only if there exists a spatiotemporal continuity of body X from t, to t and we identify A in t, with body X and B in t with body X. It means that we distinguish our friends and foes first and foremost by recalling their bodies (primarily their faces). For example, criminals are traced by looking for fingerprints or their DNA. In general, we take resemblance of body to represent resemblance of person.

However, in human beings, body transition occurs; every cell in the human body is replaced over a period of about 15 years, except for certain parts of the brain, such as the cortex. So, me-20-years-ago does not have something biological like me-today. How can self-continuation be corporal continuity if our bodies are made up many times in one lifetime? Moreover, as a counterargument, Shoemaker (1963) developed a *Gedankenexperiment* which indicated that the spatiotemporal continuity of the body is not necessary, but only the spatiotemporal continuity of the brain. Shoemaker depicts a situation as two persons A and B undergoing brain transplants surgery and ending up with each other’s brain. He concludes that although one of the brain transplants seemingly is different from the other, the brain is the main denominator in preserving the person’s continuity of identity. Thus, it appears that spatiotemporal continuity of the whole body is not necessary for personal identity, but the spatiotemporal continuity of one fragment—the brain—could suffice. If one comprehends Shoemaker’s thesis on continuity of self correctly, one supposes that each of us really exists and that we really endure through time by the brain.

Now, the formulation of the *psychological criterion* of self-continuity or what is called “the standard view” or “the dominant view” is presented. John Locke (1694/1970) is commonly considered as the founding father of this notion (Locke, 1694/1970; Parfit, 1971). This criterion is based on the concept of memory which was first proposed by Locke in the second edition of *An Essay concerning Human Understanding* (1694). Locke and his successors argue in this way that between A today and B a year ago, there exist direct memory associations if A today can remember some experiences which B had a year, or 10 years ago. For instance, I am now linked with myself yesterday and yesterday I was linked to myself of a day before and we can go so on into the past day by day. But, am I strongly linked with myself 10 years ago? This is where psychological camps of self-continuity who are aficionados of the role of memory encounter to an impasse.

There are other objections to the role of memory in persisting self-continuity. As it described earlier, they claim that self-continuity includes experiencing an unbroken sense of self over time through a higher-order mental image that is prearranged, such as a collection of memories that are self-defining or a life story. It should be noticed that memory is the sole component of personal identity over time, according to John Locke (1694/1970). Locke’s criteria, however, is considered incomplete, but in spirit appropriate. It is well known today that memory alone is not adequate.

For example, the fact that we can have intentions at one time and move them to a later time needs a kind of memory. But, no one may act upon our intentions except us, regardless of how far apart the intentions and the acts are in time. In addition, self-continuity is merely a state of affairs of memory and does not depend on memories or thoughts. To explain, sometimes when we wake up from dreams, we do not remember what we saw in the dream. But we are still confident that we have just witnessed an ongoing change from a dream. This indicates that the experiences during the dream and the experiences after waking up are fragments of the same phenomenally continuous stream of perception. Self-continuity does not, therefore, demand that the previous experience be remembered; and consequently, the function of memory is downgraded.

Later on, on the basis of that self-continuity over time, other views in philosophy extended into the continuity of the psychological structures of an individual. After John Locke’s assumption on the role of memory in persisting self-continuity, other devotees of this kind of thought were developed which are called the neo-Lockeans such as Reid (1785) and Butler (1736) as pioneers. They believe there exist “causal connections” in which mental states link together and create psychological continuation over time. But, what kinds of causal ties occur between these mental phases in order to create a continuing personal life for them? This question seems to be very complicated and they have brought more uncertainty than clarity in trying to address it.

Another philosophical doctrine (inspired from *psychological criterion*) maintains that self-continuity is defined as “overlapping chains of strong connectedness” (Noonan, 1989). If chains of linked psychological states overlap temporally—the way rope fibers overlap spatially—and if together these chains establish one “four-dimensional worm” of which every time slice is strongly linked to their direct predecessors and successors. To explain, when I remember something I did yesterday, or when the purpose I had yesterday remains and today gives rise to an action, my self-continuity has been generated by temporal chains of deep connectedness. To summarize, according to this view, psychological continuity is supposed to establish personal identity in a transitive relation: If A is continuous with B, and B is continuous with C, then A is continuous with C. In symbolic logic form, it can be depicted as: (A → B) ∧ (B → C) ∴ (A → C).

There are also some speculations among psychologists who are predominantly concerned with foundations of self-continuity. They maintain that self-continuity can be persevered through different approaches. One camp which are called “essentialists” believe that people attain self-continuity by focusing on the qualities that endure over time and trivializing changes. This approach is related to the belief that a person has an underlying and fixed essence. Self-continuity, resulting from stability of the self, correlates with the passage of time (Parfit, 1971; Lampinen et al., 2004). To put it much precisely, a person feels greater self-continuity from yesterday to today, than from a year ago to today (Peetz and Wilson, 2013). This might be due to differences in the changes between short and long temporal frames (Landau et al., 2018), or a laypeople idea that there must be more changes in a longer time frame.

Another approach which is discussed among psychologists in constructing self-continuity is through the “narration” (Chandler et al., 2003). The “narrativists” argue that individuals can create their sense of self-continuity by developing stories. We have access to diverse forms and perspectives to deal with existential problems within the cosmos of narrative. In addition, every culture is a culture of narrative, and every culture of narrative offers us an extensive repertoire of genres, models of plots, and storylines. Through narratives, we can make sense of fluctuations of life, link different experiences, and achieve meaning and consistency of the self (Becker et al., 2018). This approach underlines the connectedness between things and experiences, rather than “to imagine the existence of anything enduring or immune to time” (Chandler et al., 2003).

Others believe that there is interconnectedness mechanism between personality and cultural underpinnings of self-continuity. Some of researchers have addressed this question in a cross-sectional study in different cultures (Schmiedeck, 1979; Anderzén and Arnetz, 1999; Timotijevic and Breakwell, 2000; Becker et al., 2017). They found that members of collectivistic cultures maintain self-continuity more on an awareness of stability and associative links to one’s past, while members of individualistic cultures ground self-continuity more on stories or narratives. For example, migrants are encountered to the challenge of integration, the formation of a new identity, and the transition phase from “native” to “foreigner” in order to preserve their self-continuity in an efficient way. Besides, Erikson, 1968 and later Dien (2000) realized that identity crises arise when people lose a sense of personal consistency and historical continuity, being incapable to experience themselves as the same person they were in the past. In another study, Chandler and Lalonde (1998) hypothesized that disruption to a culture’s future or a radical breakdown from its past would pose consequences for the people. They discovered that aboriginal bands who did not effectively preserve their cultural continuity had reported suicide rates of up to 800 times the national average.

In the line of cultural aspect of self-continuity, Macmurray (1961) inspired by System Theory, tries to explain the conundrum of self-continuity by making use of sympathetic and cooperative action or confrontational opposition of persons in relation in the practices and recollections of human cultural histories.

There are some psychological assumptions which address the bases of self-continuity from a different approach. For instance, Landau and his colleagues (Landau et al., 2008) considered the question of “how self-continuity is produced.” They showed that the sense of self-continuity differs from younger and older adults, and it roots in the sense of sped up time in life. Older adults are more likely to group experiential moments or events under broader classifications, whereas younger adults are more likely to store experiential moments or events as unique classifications. When older adults look back in time, there exists only a small set of classifications to integrate (stronger self-continuity). However, when younger adults look back in time, there exists an entire set of moments or events to integrate (weaker self-continuity).

Additionally, among psychologists, some scholars argue for the role of *nostalgia* as the denominator for individuals’ perceptions of self-continuity, such that “who they are now” is linked to their sense of “who they were in the past” (Sedikides et al., 2008; Becker et al., 2018). Furthermore, according to many studies, nostalgia alleviates the pains of loneliness through pictures of social support (Zou et al., 2018), impedes boredom by replacing lost meaning in life (Van Tilburg et al., 2013), and relieves self-threat by rising self-positivity (Vess et al., 2012).

However, as a counterargument, another group believe that nostalgia has rather venomous effects: this experience can leave the person stuck in the past, and thus can alienate her from present situations by limiting the apparent range of attractive chances (Iyer and Jetten, 2011). Despite the objections, empirical study has provided consistent evidence for the role of nostalgia in preserving self-continuity. For instance, it has been indicated that people who were asked to recall nostalgic experiences report an amplified perception of continuity between their past and present selves compared with people who were requested to recollect ordinary experiences (Routledge et al., 2012; Sedikides et al., 2018). Others believe that self-continuity can be founded on the basis of dimensions that are relevant to the self, such as roles and deeds, thoughts and beliefs, social relationships, group memberships, and culture (Bluck and Alea, 2008; Chandler and Proulx, 2008; Iyer and Jetten, 2011).

Among modern psychology researchers, it seems that the role of memory has been revitalized again from its philosophical roots. They have found that the self and memory work together in creating self-continuity. Since continuity of self relies deeply on episodic memory which is endlessly storing imagery, perceptual, and sensory information about one’s ongoing experiences, they propose that memory is a key player and the self a more minor assistant in preserving chronological self-continuity (Bluck and Liao, 2013). Furthermore, a group of researchers in France and Australia (El Haj et al., 2019) discovered the role of memories which support the self-continuity in Alzheimer’s Disease. They have examined how persons with mild Alzheimer’s disease (AD) reflect on continuity of their self (i.e., whether they are the same person they were before). They conclude that people with mild AD rely on their personal and meaningful memories to maintain a continuous sense of self or even to ponder on situations in which they are concerned about their self-continuity.

Moving from the macro-level to the microlevel, in the realm of cognitive neuroscience, the neuroimaging techniques have revealed the role of the medial prefrontal cortex (MPFC) as underlying integration of self-continuity. Di Domenico and his colleagues (Di Domenico et al., 2018) found a link between (MPFC) and self-continuity persistence. In their experiment, participants see some trait adjectives and then they should respond (yes/no) to whether each trait adjective explained their past self (“Five years ago, I was …”), their present self (“At present, I am …”), and their future self (“In five years, I will be …”), while MPFC activity was scrutinized. They observed a high activity in the MPFC when responding to trait adjectives that referred to their past and future self as contrasting to their present self, indicating that the (MPFC) processes temporally separated identities in a different mode and assist in constructing self-continuity.

Hypothesizing about this enigmatic phenomenon of our existence is numerous. In fact, there are lots of conjectures and suppositions that one becomes disoriented in the forest of words which these theorists have created.

Till now, we have realized that self-continuity is required to represent both permanence and change in our existence simultaneously. Self-continuity does not mean the absence of change but encompasses a conceptual thread that is established against a backdrop of ceaseless change. In other words, self-continuity not only ensures our rightful ownership of our own memories, but also works to bind us with our own as yet unrealized future.

Furthermore, we have noticed that Identity or “self” are dynamic process—fluidlike—although its underlying nature is patchy. We are always moving through the vessel of time which gives us the sense of meaning in life. It seems that we are like sharks in the ocean of life in which our movement and continuity should be always preserved. Any inability to keep going is yet another way of dying (if it stops moving, the shark will drown). But what’s the glue, though? How is our self-continuity retained even though it is distinct in its undelaying mechanism? It is a paradox as it seems. It is a paradox whose hope for resolution has created a set of possible explanations that are so bizarre and confounding that remind us of Bertrand Russell’s quote (1959), “This is one of those views which are so absurd that only very learned men could possibly adopt them.”

## Islands of “Nowness” and the Discrete “*Self*”

For some people, passage of time is a perceptual accomplishment that provides continuity to discrete observation. But, most of what we see is a reconstruction—by filling in the incompleteness of what is perceived. The process of filling in the incompleteness of perception creates a world which appears to be complete. What we perceive of the world are illusory percepts. What we feel is not as rich in detail as it seems. In other words, consciousness is not necessarily continuous in time although it seems like that it is—its subjective side is completely different from its objective side. It is a chain of moments in which each moment comes into existence and disappears with gaps in between. Moment-by-moment replacement in the arrow of time is perceived as an illusory continuous flow (Forrest, 1995). The brain has the challenge to fill the gaps of these discrete experience of perceptual events. These discrete perceptual events can happen in different levels like sensory process, higher cognition, movement control, physiological data, speech and language, and cultural artifacts. But, one may ask whether these claims could be proven. How did we know that perception is formed in a discrete form? Why is there the smoothness of our perception of reality versus the discreetness or discontinuity of its origins at the microscopic brain level? Many psychological and neuroscientific discoveries have hinted that much of the continuity of perception is an illusion because the brain exhibits perceptual completion from a discrete form.

To answer these questions, we should notice that each of the body’s organs and biological processes have been coupled in a nested time cycle. There is a time frame for all of them: the beating of the heart, the pulsing of the breath, the cycle of the digestive system, the action of the liver, the rhythms of the sleep/awake cycle, so the brain. All of them have been fixed with a demonstration of time windows.

Besides, there exists a wealthy of experimental findings from neuroscience and psychology show that mental activities, including cognitive activities, have discrete, separated functions—they are implemented in a somewhat assigned time frame. All these assigned time frames imply to this fact that conscious perception is gappy, discrete, and bumpy and continuity is just a trick of our brain—an illusion.

By pondering in the microlevel of life, we find out that because of some neurophysiological or even survival necessity (evolutionary perspective), discrete timing of consciousness can serve better to the organism rather than a “sensibly continuous” time (Varshney et al., 2006). Varshney suggests that neurons with discrete synaptic states may function better than neurons with continuous synaptic states. In another case, Abbott and his colleagues (Abbott et al., 2016) maintained that neurons converse with one another “almost exclusively through discrete action potentials.”

Another reason for favoring discrete form of consciousness is noise tolerance (Chaudhuri and Fiete, 2016). There are many causes of noise in the brain such as sensory noise, cellular noise, motor noise. Due to these noises, information that is transported or transferred in continuous form unavoidably lose its quality and becomes corrupted. For example, let us take one of visual paths: information in human vision originates at the retina, then it is carried to the LGN *via* the optic nerve, before reaching the visual cortex (Chen and Czerwinski, 2000). Applying Shannon’s communications theory (Shannon, 1948) to communications in the brain, the presence of noise throughout such a transmission chain makes it implausible for the brain to retain continuous representation; noise will always accrue and accordingly corrupt the information, no matter how small the noise is.

Furthermore, Pöppel (1985) indicated with a neuroscientific method that how our perceptual constraints create a horizon of simultaneity—at which we distinguish audio and visual stimuli as occurring simultaneously, although the speed of sound and light are different in our physical world. We recognize audio signals as non-simultaneous when they are detached by an interval of about 6 milliseconds. If that splitting interval is shorter, we perceive audio signals as being simultaneous. Visual signals which are separated by an interval of 20–30 milliseconds are experienced as non-simultaneous. Below this threshold, they are perceived as simultaneous. In tactile impressions, the simultaneity threshold sets at approximately 10 milliseconds. According to these experiments, we perceive tactile, visual, and audio events in a temporally restricted mode. According to Pöppel, this temporally restricted structure is perceived as one “now” which are based on a clustered perception-related experiences constructed by our brains.

Evidence for discrete form of conscious perception also derives, for instance, from studies on temporal order thresholds. If subjects are requested to show the sequence of two sensory stimuli, temporal order thresholds in the domain of nearly 20 to 60 milliseconds are observed independent of the sensory modality (Hirsh and Sherrick Jr, 1961; von Steinbüchel, 1995; Wittmann and Szelag, 2003; Fink and Neubauer, 2005). To measure auditory order thresholds, for instance two clicks are sent to subject’s ears (both ears). If the stimuli are presented simultaneously, the subject will blend the stimuli perceptually so that only one stimulus is heard. A delay of one stimulus results in hearing the two clicks separately in each ear if the interval between the two surpasses 2–3 milliseconds. Although the subject hears two clicks and might even distinguish they are no longer simultaneous, she will unable to specify their temporal order correctly. The delay between the two clicks must exceed approximately 20–60 milliseconds that a subject can indicate the correct sequence. Similar threshold domains have been observed both for the visual and the tactile modality in similar experimental situations.

Another proof comes from measurements of response latencies in pursuit and saccadic eye movements. When pursuit eye movements initiate, the latency of such eye movements has a strong tendency to be within 30–40 milliseconds intervals (Pöppel and Logothetis, 1986). Similar observations have been made on saccadic eye movements for human subjects (Fuchs, 1967; Ruhnau and Haase, 1993; Tanaka and Sengco, 1997).

In short-term memory investigations, Sternberg (1966), in an experiment which is known as Sternberg task (the experiment entails memorization of a positive set, a list of items such as numbers or words) discovered that the exhaustive scanning procedure through short-term memory is disjointed, with approximate phase durations of 30–40 milliseconds.

There also exist some findings deriving from neurophysiological experiments which support the notion of discrete form of consciousness. Experiments on the brainstem auditory evoked potential prove that the midlatency response shows a clear oscillation in the first 100 milliseconds after stimulus onset (Galambos et al., 1981; Gray et al., 1989; Murthy and Fetz, 1992; Podvigin and Pöppel, 1994).

Another example is apparent movement which is debated in terms of perceptual completion. For illustration, if one visual dot is tracked in time by another further away, they seem like separate dots, separate in time. But if they are close in distance and time, the brain connects the two events by making the one dot appear to move back and forth. In this illusion, known as beta movement (important for motion pictures), the brain fills the gap and perceives a movement sensation. A similar type of illusory perceptual connection is the phi phenomenon (seen at inter-stimulus intervals greater than those associated with beta) in which a sensation of motion is experienced between the images of consecutive stimuli without a change their spatial position (Steinman, 2001). Moreover, we can find this phenomenon also in the Necker cube, which can be seen under two different perspectives (there is atemporal gap between changing two perspectives) and a phoneme sequence like CU-BA-CU-BA, where one hears either CUBA or BACU (Radilova et al., 1990).

Another piece of evidence about the discrete form of vision perception roots in what it is called in magician world visual coin trick. A magician makes the illusion of transferring a coin from one hand into the other. The magical experience of the trick happens when the closed fist of the second hand is opened and revealed to be empty. It seems according to some experiments (Beth and Ekroll, 2015), if the time interval between the false transfer and the opening of the fist increases it would be probable that the magical experience evoked by this kind of trick becomes remarkably weaker. From finding of this experiment, by increasing the temporal interval from 1 to 32 s, they observed an average reduction of the strength of the magical experience of 38%. But, how is it possible? Beth and Ekroll’s analysis make the possibility that the brain has a limit to create a perceptual completion for the intervals between discrete process of vision creation and also in seeing a stationary object.

Another proof of intermittent consciousness happens when the general anesthetic by Methohexital or Propofol is employed (usually for short surgery). This procedure results in a retrograde amnesia. Upon awakening from surgery after 5–15 min long, some patients report that what they recall is that they have being told that they will be given the drug and the next thing they recall is becoming awake and told that the surgical procedure is over. Furthermore, they were puzzled that “no time” elapsed between the two verbal events (Schwender et al., 1995).

To cover some instances of discrete form of conscious perception in the higher levels, it is worth mentioning the color phi experience (Dimmick, 1920; Kovaćs and Julesz, 1992). In this phenomenon, the mixture of spacing and timing of the two images, a person who views the wheel report a feeling of motion in the space between the two points. The first point begins to seem to be moving, and then change color sharply in the middle of its illusory path.

In behavioral levels, for instance in decision making, a person who decides to buy a product in 1.99 Euros refuse to buy the same product if priced 1 cent higher (2.00 Euros). Such a sudden change in the brain’s purchasing decision making cannot be modeled by means of a continuous representation despite broad attempts to do so (Basu, 1997). Besides, in cultural artifacts, such as hugging or shaking hand, a temporal window (around 3 s) has been observed (Schleidt et al., 1987).

So, what do all these experiments imply? From von Baer speech in 1860 at the foundation of the Russian Entomological Society in St. Petersburg about subjective time (he came up with a concept of the moment, that is, the longest possible time duration for an organism) till recent discoveries on discrete form of conscious perception, they all imply this message: underlying machinery of any organism’s cognition modalities (sight, hearing, smell, taste, and touch), there exists a temporal frame for processing (conscious perception is discrete).

Besides, for a basic understanding of human cognition, it is necessary to adopt a concept of temporal processing. By a closer look at the visual system, for instance, we realize that the integration of spatially distributed functions in the different regions of the visual cortex are determined pre-semantically by using temporal network properties rather than the content of what is processed. It is very significant to know the logistic machinery of temporal integration of spatially distributed activities in the brain prior to “what” is processed. Having explained this cognitive process, one has to differentiate strictly, with respect to the mental machinery, between two mechanisms: one pre-semantic mechanism providing a temporal frame for processing, logistical function, and the other being responsible for the content of what is processed, content function (Pöppel, 1989).

In a nutshell, we are led to the view that consciousness itself occurs in short pulses, each of which is experienced as a whole, from which it is but a short step to the view that a stream of consciousness consists of a succession of such pulses (like beads on a thread), each a short-lived total experience—“islands of nowness” (Pöppel, 1997). But, there are still unanswered, puzzling, and abrasive questions: How are these “islands of nowness,” or “specious present” connected together? How does the brain make this bumpy road as a flattened highway? What is the underlying brain mechanism (if any) for creating this illusion of continuity? What (where) is the glue of our existence?

## A Hypothesis in Search of Evidence

How those patchy, discrete, and atomic islands of nowness are interconnected together and create our smooth subjective experience? In pursuing this question, we confront to a multidimensional wonder.

Yet, the curiosity and thirst for finding an answer are the basic elements of our cognition. We inherently search for an answer in any questions of our inside and outside world. We do not stop feeling hungry. We assume that there is an answer for everything. We are born with justification and interpretation of the reality. But, there is always an answer if we keep going.

However, what is presented here is not an answer to such a profound enquiry, but a raw speculation. It is a hypothesis for search of evidence and an opportunity to comprehend, analyze and explore the foundation of self-continuity. The need to search the underpinning mechanism of the stability and connectedness of our self-continuity and its underlying neuronal machinery are the main aims.

The main focus will be specifically on the mechanism preserving self-continuity as a backbone of our identity. The hypothesis is rather eclectic because we encounter to a multifaceted phenomenon.

In brief, the hypothesis possesses the following two major principles:

1. The principle of reafference (von Holst and Mittelstaedt, 1950/1971) or corollary discharge (Sperry, 1950).
2. The principle of a time theory.

In the forthcoming section, each principle as a framework for thinking about this deep concept of our being—the continuity of self will be explained. I do it humbly and nervously. I try to add to the story previously generated by this pantheon of scholars and scientists. It seems to me it is frightening, to say the least.

## A Compass for Future Directions

Change is the law of nature, but for many survival and sociological reasons, we should maintain our identity in the passage of time. A person should be able to think that he or she is the same person now as he or she was a few moments ago. Giving a poetic example, we need to be able to point to the red apple and say, “It’s the same apple as green,” to be able to say that an apple has shifted from green to red. This situation is sometimes called the principle of the preservation of identity. But persistence is not restricted to short period and it also applies for long-term situations (e.g., years.). Someone seems like the same person today as while he or she was younger years before. We need to persist and never vanish from our identity and selves within a block of space time. It seems that this perception of persistence is inseparably related to the phenomenon of time. It seems that persistence and time are two sides of the same coin.

We appear to maintain our identity vehemently in the arrow of past/present/future. However, the concept of persistence is not restricted to our identity. There is so-called *object persistence* in which despite an alteration in either its spatiotemporal state or its structure, a person tends to perceive an entity as being the same. For instance, in the *classic tunnel effect* (a car going through a tunnel), when motion is hidden, perceptual continuity is retained by amodal completion. That is, the spectator has an experiential phenomenon in which, as the same object, the object which entered the tunnel undeniably passed through and left.

In addition, the audience also believes cognitively that it is the same car, even though the color of the exiting car is significantly different from the original one. The impulsive pattern of persistence is so overwhelming that the observer thinks that a small modification may have occurred inside the tunnel in some way. There are lots of experiments have done and many of them concluded that object persistence happens because of the principle of spatiotemporal priority (Michotte, 1991; Flombaum and Scholl, 2006).

Another experimental example of the object persistence is a golf ball changing colors as it moves across. Even if it turns into a strawberry just as it falls into the hole, the golfer still considers it to be the same ball (his or her ball) that for unexplained reasons changed drastically. The brain does not reflect the possibility that the ball vanished and was replaced by a strawberry (Gruber and Block, 2013).

Putting aside the concept of persistence for moment, in the previous section it was argued that the relationship of time and self is a significant factor and that self-consciousness is inseparably associated with our precise time consciousness. A few scholars, however, have discussed the phenomenology of altered perception of time in patients suffering from mental disorders. We experience ourselves in the rhythm of world time when exploring the world. Though it looks trivial, it is a notable fact that experiencing reality is only possible through experiencing the “now”; the current state.

Turning back to the hypothesis, in order to preserve self-continuity, two major principles should be followed: *The principle of reafference (or corollary discharge)* and *The principle of a time theory*. It is assumed that if these two principles function properly, the self-continuity is maintained. Firstly, what is the principle of reafference (or corollary discharge) and how does it function in preserving continuity?

In brief, the principle of reafference or corollary discharge (to the best of knowledge, von Holst & Mittelstaedt’s principle of reafference and Roger Sperry’s corollary discharge proposed independently and almost simultaneously (both published in 1950/1971) and they both talked about somehow the same concept) is on the basis of three main factors: *anticipation*, *comparison (or match)* and *image storing*. According to these principles, there is always a copy of the outside world (EK or efference copy) in the CNS which works as what is called a *compass image* in order to be compared with the main goal in an anticipatory form. This copy of the outside world (*compass image*) is preserved in *memory*. Now, there is information about the past in the memory which is useful only (according to the principle of reafference) to the extent that it allows the organism to *anticipate* what may happen in the future.

When the action (or feeling) is spatially and temporally matched as expected (matched with the compass(stored)image/copy), the brain interprets the sensation as self-generated. If there is a mismatch, if the signals are spatially and temporally discordant with self-touch, you decree it as being done by another agent. In this respect, memory did not evolve to allow us to recall about the past, tell our long-ago life stories, and enjoy sharing it. The unique evolutionary function of memory is to allow organisms to predict what will occur, when it will occur, and how to best react when it does occur.

To put it simply, an example might help more: we draw on previous experiences in our everyday life activities to envision and simulate episodes that could occur in our personal futures. For example, when we envision various ways of tomorrow, a date or a vacation, we project ourselves into the future based on what we remember from the past. Indeed, in order to predict what will happen in the future, we use knowledge about the past.

Or, consider this situation: when we plan, we expect that it occurs at a specific time and location in space. You plan to drink a glass of water, you expect to drink to quench your thirst. When the plan is spatially and temporally matched as expected (according to the principle of reafference, as you remember, matched with the copy/image), your brain interprets the sensation as self-generated. If there is a mismatch (with the copy/image), if the signals are spatially and temporally discordant with the plan, you declare it as being done by another agent.

So, how could the reafference principle be applicable in this hypothesis? CNS not only does have a copy *(compass image)* of the outside world, also it has an inclusive image of the *self—*the identity of an individual. There seems to be a general understanding that an inner representation of the external world of man, involving himself at the core, exists. There is a consensus among experts that each person has one such system, a coherent type of internal structural representations, without which actions can hardly be structured in a coherent manner. This copy of self *(or inclusive image)* which is comprised of not more than hundred images of lifetime events are stored as an autobiographical record in our episodic memory. These 100 images also are activated when we time travel to our personal past. Meanwhile, Ernst Pöppel did an experiment (he mentioned this experiment in personal communication) several years ago with this research question: how many images in our mind are activated when we time travel in our personal past? Several hundred people take part in this experiment from different age groups, different professions, and members of different cultures. Time travel experience of these people’s past showed that everyone can only activate a few hundred images, although we may suppose that it may be more.

Having said about the *inclusive image* which is stored in CNS, our brain consonantly compares *(comparator mechanism)* this image with different contexts in the outside world in an anticipatory system in order to make the *self* as a functional unity in a socially and historically fabricated form (the brain always desires to categorize and compare). The brain, is not a passive recipient of sensory information from world outside. It is now thought that our brain works like an anticipatory machine and it actively predicts sensations (Picard and Friston, 2014). In other words, CNS constructs a model of the environment (outside world) and compares the *inclusive image* to this model. When this inclusive image matches with the context “outside,” thus matching occurs between the reality of consciousness (the subject) and a reality outside of consciousness (the outside context).

So, when the comparator mechanism functions efficiently, the *Self* instantiates a wide variety of behavioral and mental circumstances preferred by the perceived needs of the sense in which it is expressed and in accordance with them. In a normal situation, when the image (copy of self) is in concert with the goals of outside world, we are not so aware of them. We continue our normal life. But, when the situation becomes turbulent, we know that something is wrong. For example, when I meet my mentor in the lab, I should maintain a completely different self-continuity (identity) from when I meet my girlfriend in a bar. Thus, upon visiting my mentor, the brain begins comparing the stored *inclusive image*s (comprised of images of myself and his) with the external world (the image of him who sat in front of me) in an anticipatory mechanism. Then, my self-continuity functions properly because there is a match between what is seen in the extremal world and the stored inclusive images in the CNS. So far, so good. But, this is not the whole story.

Having mentioned the anticipatory mechanism of the brain, as a side remark, it should be remembered that our brain appears to intersect the arrows of time. In fact, our common sense perceives the arrow of time as *past–present–future*. But, new investigations on our understanding of the psychological time appears that the arrow is *future–present–past*. The brain anticipates the “now,” and it asks always: “what is next?” (Pöppel, 2009). Remarkably, in this sense, time theory begins from the future (in Heidegger’s analogy; Heidegger, 1927/2002), from the conceptualization of “what’s next?.”

Our brain is a “significance detector device” (SDD) in a sense that it is able to find—within an enormous amount of noises in the outside world—a particular desire which seems “meaningful” or better to say satisfying in an anticipatory way. The brain—as a multisystem parallel processor—creates the anticipatory pattern or *(SDD)* by interacting with different more or less independent “modules” in its scattered regions.

The powerful intuitive persuasion of self-continuity is maintained only by the benefit of this system which is extremely in an efficient operation. When the rhythms of the inner world no longer fit the dynamics of the reality we experience, the dignity of our self-image disintegrates. In other words, any failure to such a mechanism leads to various kinds of internally and externally generated turbulences. For instance, if we take schizophrenia as a disorder of the self (Cutting, 1990), then there should be a turbulence between the original copy of self and its comparator mechanism with the outside world. The lack of compatibility between the *inclusive image* and the outside world or an unbroken band between the self and the other leads to a catastrophe. Or consider, for instance, the TOT (tip of tongue) situation. A TOT happens when there is a sense that a piece of information is known, but it is not available for conscious report. As you see, there is a conflict between inside and outside world. In other words, there is a piece of information that cannot be matched with the outside context of conversation. There are lots of these situations which should be observed.

It should be noted that the principle of reafference has been applied by many other researchers in order to explain the higher-order functions of human. The model of reafference principle has been also employed in understanding of behavioral control and also modeling human behavior by others. For example, Tanida and Pöppel (2006) elucidate hierarchical model of operational anticipation windows in driving an automobile on the basis of the reafference principle and experimental results on temporal perception and cognitive control. Furthermore, Glimcher (2003) refers explicitly to the reafference principle to explain human’s economic behavior and how it might be embedded within neuronal programs. In another case, Merker (2005) posits the principle of reafference into the center of his reasoning in his theory of consciousness. In much more interesting case, Lindner and his collaborators (Lindner et al., 2005) diagnosed in schizophrenic patients a high association between measures of oculomotor control and disorders of agency. In their experiment, they found out that the poorer the EK (efference copy) was represented during an oculomotor task, the higher were the problems in the expression of self-reference in such patients. Likewise, there lots of evidence for efference copy failure is strictly linked with hallucinations and delusions in both schizophrenia and bipolar patients (Gould, 1948; Feinberg, 1978; Blakemore et al., 2002; Ford and Mathalon, 2005; Leube et al., 2010).

Now, let us turn to the second principle of the hypothesis—*the principle of a time theory*. In the first place, why does the hypothesis need a time theory? Why is there an emphasis on the importance of time in this hypothesis? The answer lies in the fact that the structures of behavior in any organism tend to be predominantly temporal. Take a close look at motions such as walking, for instance. Or, consider the way we communicate with each other. Any temporal change in an utterance, though the sequence of phonemes may remain unchanged can create a different impression on the addressee. In all of our activities, temporal aspects are crystal clear. Surprisingly, psychologists and neuroscientists have demonstrated reluctance to infer the existence of such an important factor like time in an organisms’ behavior.

It is also important for the brain, in line with this claim, to know not only where to place focus, but when. From neural computation to driving a car to playing the piano, timing is the denominator of almost every action we participate in. In addition, recognizing when something happens, intuitionistically, it allows us to focus energy to change our actions at that expected point in time.

Furthermore, what the principle of reafference (also the corollary discharge) lacks is availability of a theory of time or better to say precise real-time system. Although von Holst and Mittelstaedt (1950/1971) talk about “*a certain temporal delay, which is stored into the neighboring ganglia,”* they do not elucidate what they exactly mean by that *delay* in the principle of reafference. They do not mention how much that temporal delay takes or any clue about a clear timing system. Therefore, the reason why the importance of time in the reafference principle is emphasized lies in the fact that the underlying process should have a crucial timing.

Let us focus on a specific example: it needs time-referencing conditions to perceive something, such as perceiving a pen, and saying “Here is a pencil.” In other words, not only is the pencil present to us, that is, very fixed to our senses, but we also have to realize that it has maintained the identity of being-this-pencil for a certain period of time, be it 2 days or just two seconds. If the pencil appears one moment and the next disappears, or if it becomes a desk and then a stack of books, then we can never claim “Here is a pencil.” As (Kant, 1908) called “synthesis of identification in a term,” that is, there is one thing for us that means that over time we find a unity in its appearances, so that we can name it with a word and endorse its existence as well.

It is a fact that the reality of time is the foundation of consciousness. So, due to fact that our perception has a temporal character, dysfunction in the temporal system brings lots of problems for an organism.

Therefore, a timing system to the principle of reafference in order to elucidate the hypotheses and accordingly the conundrum of self-continuity seems necessary. According to the concept of the reafference principle, humans and other organisms as well build up an internal representation, a model of the universe, a schema, a cognitive map, or what was called earlier *compass image* from the environment in which they have located. Our brain constantly checks the compass image with what we plan to do. That is, our brain relentlessly seeks in an anticipatory form a cognitively compatible relationship with the objective world by comparison. For instance, consider preparing a meal. We have agreed and allocated a certain amount of time for that activity. Naturally, if it has become our normal everyday action, such a decision will remain implicit or unconscious for us. We assign a target and to accomplish that effectively, the behavioral regulation neural system has been programmed in a way that monitors each of our actions in a serial order. Furthermore, our brain constantly checks the internal goal (e.g., preparing a meal) with reaching the goal. There is always a precise timing between comparator mechanism and anticipation system. In other words, if the precise timing adjustment (or temporal integration) between comparator mechanism and anticipation system encounters difficulty, because of some illnesses, for instance, there is a temporary imbalance in the process. This internal, timeless disorganization brings about a crisis. As a result, the crisis affects the cognitive-emotional structure of the brain and requires rebalancing in schizophrenic patients, for example. Lack of cognitively compatible relationship with outside world (disorganized precise timing in comparator and anticipatory mechanism) causes a while new self when a new time appears.

So, according to this hypothesis, there exists a precise timing system between comparator and anticipatory system which synchronizes all of the functions in preserving self-continuity. Among many other factors, this precise timing system is a crucial feature and leading denominator with which self-continuity is flourished. To recap, there should be a *temporal precision regulator (TPR)* between CNS and peripheral world to grasp the outside world—when this temporal precision regulator becomes dysfunctional, many problems occur.

As a matter of fact, predicting events in time is intrinsic to our mental life, and plays a leading role in the temporal structure of consciousness. Indeed, that precise timing system of comparator and anticipatory system of the brain help to bridge isolated events together and to shape the sense of continuity (a semantic glue) that constitutes our subjective life. But, what could it be that precise timing regulator? What is the *temporal precision integration system* with which all perceptions (not only enjoying our self-continuity) functions properly?

To answer this profound question, the precise timing system may lie in what Ernst Pöppel calls “the windows of presence” with duration of approximately 2–3 s or “primordial events” with duration of 30–40 milliseconds. According to his theory, this 2- to 3-s time horizon or 30–40 milliseconds provide a temporal stage on which any conscious activity is represented. So, anticipatory system of the brain always asks “What is new in the world?” (Pöppel, 2009). From this view, these temporal windows are necessary (not sufficient) neuronal machinery for the creation of our identity, perception, emotions, memories, and thoughts.

Then, what could be the sufficient condition? The sufficient condition lies in the comparator and anticipatory mechanism of our brain on the basis of the principle of reafference. I believe that by merging time theory of 2–3 s (2–3 s windows)/30 to 40 milliseconds and the principle of reafference’s comparator and anticipatory mechanism of the brain, we pave the road for decoding the underlying basis of our self-continuity.

As the closing remarks, the hypothesis which was presented here could not be the ultimate answer to this enigma. Instead, it is only an endeavor to show that there is always a missing link which most of us ignore as a scientific scotoma and trap into a myopic scientific view. In fact, what was explained here could be useful as a complementary factor for any theory about self-continuity.

## Author contributions

The author confirms being the sole contributor of this work and has approved it for publication.

## Funding

The author gratefully acknowledge the financial support of Hanns-Seidel-Stiftung without which the present study could not have been completed.

## Conflict of Interest

The author declares that the research was conducted in the absence of any commercial or financial relationships that could be construed as a potential conflict of interest.

## Publisher’s Note

All claims expressed in this article are solely those of the authors and do not necessarily represent those of their affiliated organizations, or those of the publisher, the editors and the reviewers. Any product that may be evaluated in this article, or claim that may be made by its manufacturer, is not guaranteed or endorsed by the publisher.
